# Comparison of strain and shear wave elastography for qualitative and quantitative assessment of breast masses in the same population

**DOI:** 10.1038/s41598-018-24377-0

**Published:** 2018-04-18

**Authors:** Hyo Jin Kim, Sun Mi Kim, Bohyoung Kim, Bo La Yun, Mijung Jang, Yousun Ko, Soo Hyun Lee, Heeyeong Jeong, Jung Min Chang, Nariya Cho

**Affiliations:** 1 Department of Radiology, Seoul National University Bundang Hospital, Seoul National University College of Medicine, 82, Gumi-ro 173 Beon-gil, Bundang-gu, Seongnam-si, Gyeonggi-do, Korea; 2 Division of Biomedical Engineering, Hankuk University of Foreign Studies, Oedae-ro 81, Mohyeon-myeon, Cheoin-gu, Yongin-si, Gyeonggi-do, Korea; 3 Department of Radiology, College of Medicine, Chungbuk National University, 776 1sunhwan-ro, Seowon-gu, Cheongju, Korea; 4 Department of Health Promotion, Seoul National University Bundang Hospital, 82, Gumi-ro 173 Beon-gil, Bundang-gu, Seongnam-si, Gyeonggi-do, Korea; 5 Department of Radiology, Seoul National University College of Medicine, 101 Daehakro, Jongno-gu, Seoul, Korea

## Abstract

We investigated addition of strain and shear wave elastography to conventional ultrasonography for the qualitative and quantitative assessment of breast masses; cut-off points were determined for strain ratio, elasticity ratio, and visual score for differentiating between benign and malignant masses. In all, 108 masses from 94 patients were evaluated with strain and shear wave elastography and scored for suspicion of malignancy, visual score, strain ratio, and elasticity ratio. The diagnostic performance between ultrasonography alone and ultrasonography combined with either type of elastography was compared; cut-off points were determined for strain ratio, elasticity ratio, and visual score. Of the 108 masses, 44 were malignant and 64 were benign. The areas under the curves were significantly higher for strain and shear wave elastography-supplemented ultrasonography (0.839 and 0.826, respectively; *P* = 0.656) than for ultrasonography alone (0.764; *P* = 0.018 and 0.035, respectively). The diagnostic performances of strain and elasticity ratios were similar when differentiating benign from malignant masses. Cut-off values for strain ratio, elasticity ratio, and visual scores for strain and shear wave elastography were 2.93, 4, 3, and 2, respectively. Both forms of elastography similarly improved the diagnostic performance of conventional ultrasonography in the qualitative and quantitative assessment of breast masses.

## Introduction

Ultrasound (US) elastography is a complementary imaging technique for characterizing breast lesions^1–5^; when combined with conventional B-mode ultrasound imaging, it increases diagnostic performance^2–7^. There are two types of elastography: strain and shear wave elastography. Strain elastography applies compressive force to breast tissue and measures lesion stiffness. Generally, malignant lesions are harder than benign lesions^2–4,6–8^. Lesion stiffness is expressed on a colour scale for qualitative assessment and/or expressed as lesion-to-fat strain ratio (i.e., “strain ratio”) for semi-quantitative assessment. For a colour scale, the five-point visual scoring system invented by Tsukubas is used^1^. Depending on the imaging system used, a colour closer to either the blue or red end of the spectrum indicates increased stiffness and an increased probability of malignancy. The strain ratios of malignant lesions are greater than those of benign lesions^1^. Shear wave elastography exploits the fact that shear waves propagate faster in hard than in soft tissue; this can be qualitatively assessed by analysing a colour-scaled image and/or quantitively assessed by determining the maximum elasticity value (kPa) or the mean mass ratio of elastic to fatty tissue (i.e., “elasticity ratio”). A colour closer to the red end of the spectrum represents a higher kPa value, or greater elasticity ratio, indicating a malignant lesion^5^.

Many studies have shown that combining one of these two elastographic techniques with conventional ultrasonography improves differentiation between benign and malignant breast masses^2–4,9^. However, direct comparison of both types of elastography in the same population is relatively rare^10–12^; to the best of our knowledge, few studies have compared the strain and elasticity ratios achieved with these techniques^11,12^. Additionally, regarding scoring colour-scale images obtained by strain and shear wave elastography, and the strain and elasticity ratios, cut-off values dividing benignity and malignancy differ among published studies^5,8,13^. Our study aimed to compare the added diagnostic benefits of two types of breast ultrasound elastography when combined with conventional ultrasonography, and to determine the optimal cut-off points of strain and elasticity ratios, and visual scores for colour-scale images from two types of elastography, in differentiating between benign and malignant breast lesions.

## Results

### Lesion characteristics

Of 108 masses, 44 were malignant and 64 were benign. Histopathological results of the lesions are summarized in Table 1. Among the 44 malignant masses, 42 were confirmed as malignant and two were upgraded to a diagnosis of ductal carcinoma *in situ* (DCIS) after atypical ductal hyperplasia was confirmed with US-guided biopsy. All malignant masses underwent surgery for treatment. The most common malignant and benign masses were invasive ductal carcinoma and fibroadenoma, respectively. Among the 64 benign lesions, four papillomas, one phyllodes tumour, and one fibroadenoma were excised. The remaining benign lesions underwent follow-up examination for >12 months and showed no change.

**Table 1:** Histopathological results of biopsied lesions.

Histopathological result	Lesions, n
Malignant	
Invasive ductal carcinoma	28
Ductal carcinoma *in situ*	14
Invasive lobular carcinoma	1
Mucinous carcinoma	1
Total	44
Benign	
Fibroadenoma	25
Intraductal papilloma	8
Fibroadenomatoid change	7
Mammary duct ectasia	6
Fibrocystic change	4
Complex fibroadenoma	2
Other benign lesions*	12
Total	64

^*^Other benign lesions: pseudoangiomatous stromal hyperplasia, nodular adenosis, sclerosing papilloma, benign phyllodes tumour, post-mammotome change, suture granuloma, inflamed granulation tissue, foreign body and foreign body reaction, fibrous mastopathy, ruptured epidermal cyst, fibroadipose tissue, and no diagnostic abnormality.

The mean size of mass was 1.17 cm (range, 0.4–2.4 cm). The mean depth of the lesion measured as the distance from the skin to the centre of the mass was 1.11 cm (range 0.40–2.20 cm). Initial BI-RADS categories at biopsy were 4a, 4b, 4c, and 5 in 74, 14, 4, and 15 masses, respectively.

### Diagnostic performance

In ROC analysis, there was a statistically significant difference between using conventional images alone (area under the curve [AUC], 0.764; 95% CI, 0.687–0.841) and using conventional images plus colour-scale images with the cancer probability scale (with strain elastography; AUC, 0.839; 95% CI, 0.768–0.910; *P* = 0.013; with shear wave elastography; AUC, 0.826; 95% CI, 0.760–0.892; *P* = 0.035). The difference was not significant, however, when comparing conventional plus strain and conventional plus shear wave images (*P* = 0.656).

**Figure 1:**
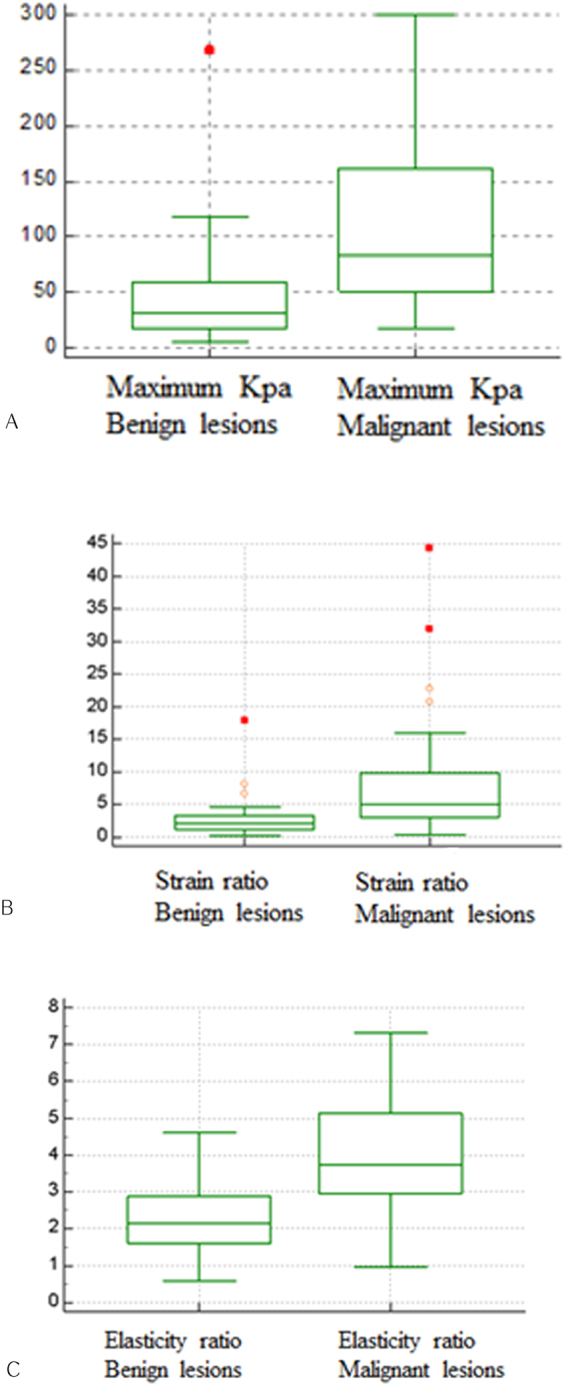
The maximum kPa values, strain ratios, and elasticity ratios of benign and malignant breast lesions. (**A**) The mean maximum kPa values of benign and malignant lesions were 43.24 ± 40.11 and 105.47 ± 73.12, respectively. (**B**) The strain ratios of benign and malignant lesions were 2.275 ± 0.998 and 3.936 ± 1.666, respectively. (**C**) The elasticity ratios of benign and malignant lesions were 2.576 ± 2.443 and 8 ± 8.55, respectively.

**Figure 2:**
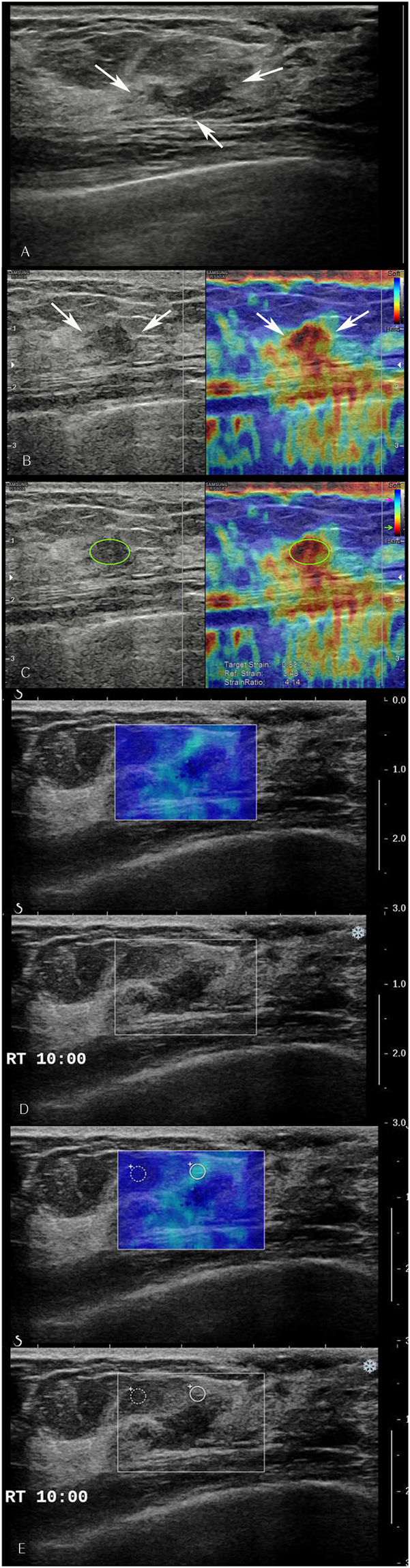
An abnormality detected via screening mammography in a 50-year-old woman. (**A**) Grayscale ultrasound shows an indistinct irregular isoechoic mass (arrows) in the 10 o’clock area of the right breast which was categorized as a category 4a tumour with low suspicion of malignancy. (**B**) By strain elastography, the mass was graded with a visual score of 5 according to the Tsukuba system (arrows). (**C**) Strain ratio was 4.1. (**D**) By shear wave elastography, the mass was graded with a colour score of 3. (**E**) Elasticity ratio was 5.41. Ultrasound core biopsy confirmed the tumour to be a ductal carcinoma *in situ*.

**Figure 3:**
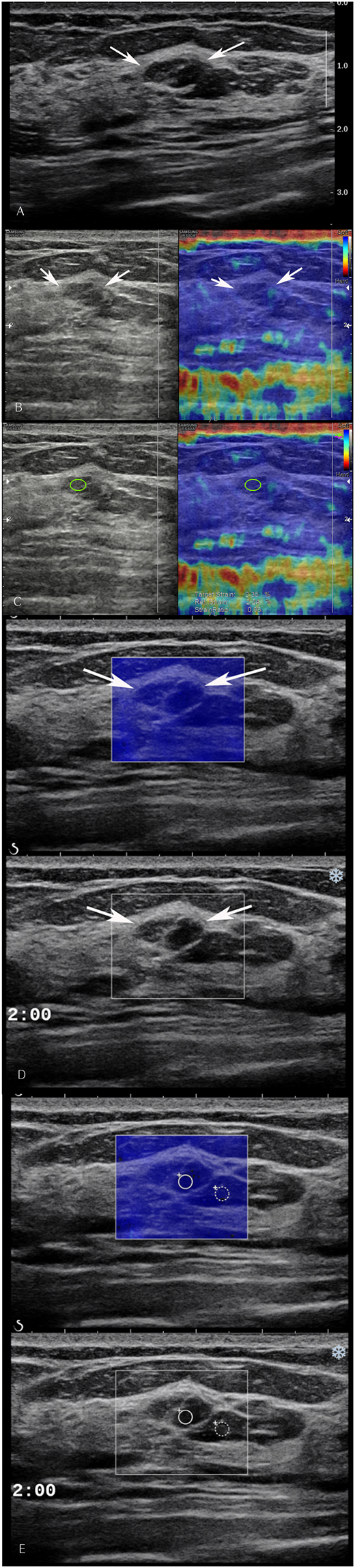
An abnormality detected via screening mammography in a 51-year-old woman. (**A**) Grayscale ultrasound shows an indistinct oval heterogeneous echoic mass (arrows) in the 2 o’clock area of the left breast which was categorized as a category 4a tumour with low suspicion of malignancy. (**B**) By strain elastography, the mass was graded with a visual score of 1 according to the Tsukuba system (arrows). (**C**) Strain ratio was 0.9. (**D**) By shear wave elastography, the mass was graded with a visual score of 1. (**E**) Elasticity ratio was 1.4. Ultrasound core biopsy confirmed the tumour to be a fibroadenoma.

For benign and malignant lesions, the mean strain ratios were 2.275 ± 0.998 and 3.936 ± 1.666, respectively (*P* < 0.001), and the mean elasticity ratios were 2.576 ± 2.443 and 8 ± 8.55, respectively (*P* < 0.001). The mean maximum kPa values for benign and malignant lesions were 43.24 ± 40.11 and 105.47 ± 73.12, respectively; mean kPa values were 36.27 ± 33.94 and 88.25 ± 61.13, respectively; and minimum kPa values were 27.69 ± 27.7 and 64.66 ± 45.33, respectively, using shear wave elastography (all *P* < 0.001; Figs 1–3).

The cut-off values were 2.93 (sensitivity, 77.3%; specificity, 78.1%; AUC, 0.800; 95% CI, 0.713–0.871) for strain ratio, 4 (sensitivity, 65.9%; specificity 89.1%; AUC, 0.810; 95% CI, 0.723–0.879) for elasticity ratio, and 36.1 (sensitivity, 90.9%; specificity, 56.2%; AUC, 0.809; 95% CI, 0.722–0.879) for kPa. The 95% CI derived from the conventional images for multi-readers was lower than those derived from the cut-off values of the strain and elasticity ratios, and kPa. Furthermore, when additionally reviewing colour-scale images with conventional images, the 95% CI was higher than those derived from the cut-off values of the strain and elasticity ratios, and kPa.

### Interobserver agreement

Regarding the visual score for colour-scale elastography images, the interobserver agreement was fair on strain elastography and good on shear wave elastography (Table 2). Regarding BI-RADS grading, the interobserver agreement increased from fair to moderate upon combining shear wave elastography with conventional ultrasonography. Upon combining strain elastography with conventional ultrasonography, the kappa value increased, but remained within the category of “fair” (Table 2). Regarding the cancer probability scale, the interobserver agreement was excellent even without addition of elastography, although the ICC values increased upon adding elastography (Table 3).

**Table 2:** Interobserver agreement of visual score grading by strain and shear wave elastography and the Breast Imaging Reporting and Data System categorization.

Modality	Kappa (strength of agreement)	95% confidence interval
Visual score		
Strain elastography	0.281 (fair)	0.257–0.350
Shear wave elastography	0.681 (good)	0.619–0.727
Breast Imaging Reporting and Data System		
Conventional US alone	0.246 (fair)	0.220–0.276
Conventional US + strain elastography colour images	0.307 (fair)	0.270–0.395
Conventional US + shear wave elastography colour images	0.524 (moderate)	0.469–0.558

**Table 3:** Interobserver agreement of cancer probability grading.

Modality	Intraclass correlation coefficient (reliability)	95% confidence interval
Conventional US	0.867 (excellent)	0.807–0.908
Conventional US + colour-scaled strain images	0.928 (excellent)	0.902–0.949
Conventional US + colour-scaled shear wave images	0.964 (excellent)	0.951–0.974

### Hypothetical performance according to cut-off value

On strain elastography, when lesions were downgraded from BI-RADS category 4a to 3 hypothetically by a colour score <3, diagnostic performance was the best; specificity increased significantly without significant loss of sensitivity in all four readers (Table 4). On shear wave elastography, when lesions were downgraded from BI-RADS category 4a to 3 hypothetically by a colour score <2, diagnostic performance was the best; specificity increased significantly without significant loss of sensitivity, except in reader 3 (in whom the sensitivity decreased significantly; Table 4). When lesions were downgraded from BI-RADS category 4a to 3 hypothetically by the cut-off of strain ratio (≤2.93), specificity increased significantly without a significant loss of sensitivity, except in reader 3 (in whom the sensitivity decreased significantly; Table 4). When lesions were downgraded from BI-RADS category 4a to 3 hypothetically by the cut-off of elasticity ratio (≤4), specificity increased significantly, but with significant loss of sensitivity in three readers (Table 4). When lesions were downgraded from BI-RADS category 4a to 3 hypothetically by the cut-off of kPa (≤36.1), specificity increased significantly without significant loss of sensitivity in all four readers (Table 4).

**Table 4:** Diagnostic performance of pre-adjusted and post-adjusted Breast Imaging Reporting and Data System (category 4a downgraded to category 3).

Reviewer	Sensitivity, % (n)		Specificity, % (n)	
	Pre-adjusted	Post-adjusted	Pre-adjusted	Post-adjusted
Shear wave elastography, colour score, cut-off <3				
1	93.2 (41/44) [85.7–100]	86.4 (38/44) [76.2–96.5]	14.1 (9/64) [5.5–22.6]	43.8 (28/64) [31.6–55.9]
2	97.7 (43/44) [93.3–100]	95.5 (42/44) [89.3–100]	12.5 (8/64) [4.4–20.6]	21.9 (14/64) [11.7–32.0]
3	95.5 (42/44) [89.3–100]	90.9 (40/44) [82.4–99.4]	12.5 (8/64) [4.4–20.6]	34.4 (22/64) [22.7–46.0]
4	97.7 (43/44) [93.3–100]	93.2 (41/44) [85.7–100]	7.8 (5/64) [1.2–14.4]	39.1 (25/64) [27.1–51.0]
Mean $\pm$ SD	96.0225 ± 2.17858	91.4750± 3.88116	11.7175 ± 2.70681	34.8925 ± 9.40122
Shear wave elastography, colour score, cut-off <2				
1	93.2 (41/44) [85.7–100]	86.4 (38/44) [76.2–96.5]	14.1 (9/64) [5.5–22.6]	62.5 (40/64) [50.6–74.4]
2	97.7 (43/44) [93.3–100]	97.7 (43/44) [93.3–100]	12.5 (8/64) [4.4–20.6]	40.6 (26/64) [28.6–52.7]
3	95.5 (42/44) [89.3–100]	79.5 (35/44) [67.6–91.5]	12.5 (8/64) [4.4–20.6]	54.7 (35/64) [42.5–66.9]
4	97.7 (43/44) [93.3–100]	90.9 (40/44) [82.4–99.4]	7.8 (5/64) [1.2–14.4]	43.8 (28/64) [31.6–55.9]
Mean ± SD	96.0225 ± 2.17858	88.6375 ± 7.65087	11.7175 ± 2.70681	50.3925 ± 10.07454
Strain ratio, cut-off ≤2.93				
1	93.2 (41/44) [85.7–100]	81.8 (36/44) [70.4–93.2]	14.1 (9/64) [5.5–22.6]	70.3 (45/64) [59.1–81.5]
2	97.7 (43/44) [93.3–100]	95.5 (42/44) [89.3–100]	12.5 (8/64) [4.4–20.6]	48.4 (31/64) [36.2–60.7]
3	95.5 (42/44) [89.3–100]	79.5 (35/44) [67.6–91.5]	12.5 (8/64) [4.4–20.6]	60.9 (39/64) [49.0–72.9]
4	97.7 (43/44) [93.3–100]	88.6 (39/44) [79.3–98.0]	7.8 (5/64) [1.2–14.4]	46.9 (30/64) [34.6–59.1]
Mean ± SD	96.0225 ± 2.17858	86.3650 ± 7.18364	11.7175 ± 2.70681	56.6425 ± 11.07334
Elasticity ratio, cut-off ≤4				
1	93.2 (41/44) [85.7–100]	77.3 (34/44) [64.9–89.7]	14.1 (9/64) [5.5–22.6]	78.1 (50/64) [68.0–88.3]
2	97.7 (43/44) [93.3–100]	95.5 (42/44) [89.3–100]	12.5 (8/64) [4.4–20.6]	56.3 (36/64) [44.1–68.4]
3	95.5 (42/44) [89.3–100]	77.3 (34/44) [64.9–89.7]	12.5 (8/64) [4.4–20.6]	65.6 (42/64) [54.0–77.3]
4	97.7 (43/44) [93.3–100]	84.1 (37/44) [73.3–94.9]	7.8 (5/64) [1.2–14.4]	46.9 (30/64) [34.6–59.1]
Mean ± SD	96.0225 ± 2.17858	83.5200 ± 8.57855	11.7175 ± 2.70681	61.7225 ± 13.35069
Shear wave elastography, maximum kPa, cut-off ≤36.1				
1	93.2 (41/44) [85.7–100]	86.4 (38/44) [76.2–96.5]	14.1 (9/64) [5.5–22.6]	59.4 (38/64) [47.3–71.4]
2	97.7 (43/44) [93.3–100]	95.5 (42/44) [89.3–100]	12.5 (8/64) [4.4–20.6]	45.3 (29/64) [33.1–57.5]
3	95.5 (42/44) [89.3–100]	86.4 (38/44) [76.2–96.5]	12.5 (8/64) [4.4–20.6]	48.4 (31/64) [36.2–60.7]
4	97.7 (43/44) [93.3–100]	90.9 (40/44) [82.4–99.4]	7.8 (5/64) [1.2–14.4]	40.6 (26/64) [28.6–52.7]
Mean ± SD	96.0225 ± 2.17858	89.7700 ± 4.35194	11.7175 ± 2.70681	48.4400 ± 7.96820

SD, standard deviation; Except for Mean ± SD values, data are percentage (number of lesions/total) [95% confidence interval CI].

## Discussion

In this study, we directly compared strain and shear wave elastography in the same patients using qualitative and quantitative assessment. The diagnostic performance using conventional images plus colour-scale images of both types of elastography was superior to that of using conventional images alone. Interobserver agreement between four radiologists for visual scale grading was better on shear wave than strain elastography. The strain and elasticity ratios were associated with similar diagnostic performances, which were superior to that of using conventional images alone, in the context of differentiating between benign and malignant breast masses with cut-off values of >2.93 and >4 for fat strain and elasticity ratios, respectively. When lesions were downgraded from BI-RADS category 4a to 3 hypothetically by a strain elastography colour score <3, or by the cut-off of kPa (≤36.1), specificity increased significantly without significant loss of sensitivity.

Little published data pertaining to the direct comparison of diagnostic performance between strain and shear wave elastography in the same patients exists^11,12,14^. In this study, the difference in diagnostic performance was not significant when comparing conventional plus strain images and conventional plus shear wave images. Some previous studies reported that the joint use of US and shear wave images showed no statistically significant improvement over the use of US alone^15^. However, our finding of improved diagnostic performance with colour-scale images of elastography was consistent with previous studies on the combined use of conventional images plus colour five-point scoring^5,6,16^.

Interobserver variability affects the diagnostic performance of elastography for breast tumour characterization. Interobserver agreement in grading visual score has previously been reported as moderate^17,18^. In our study, the interobserver agreement between the four radiologists in visual score was better on shear wave than strain elastography. In grading BI-RADS, interobserver agreement increased from fair to moderate when combining shear wave elastography with conventional ultrasonography; conversely, the combination of strain elastography and conventional ultrasonography was associated with improved, though still only fair, interobserver agreement. In grading cancer probability, interobserver agreement was excellent whichever tested modality was used, but the ICC values increased with the addition of elastography.

Few previous studies have compared strain and elasticity ratios, as determined by strain and shear wave elastography, of benign and malignant breast tumours in the same patients^11,12^. This study found the mean strain ratio and elasticity ratios to be significantly different between benign and malignant lesions in both strain elastography and shear wave elastography. Previous studies have compared the strain ratio and five-point scoring system^9,19^, strain ratio and conventional US^9^, and between quantitative shear wave elastography parameters, and have found that the strain and elasticity ratios exhibit similar or superior diagnostic performance compared to visual scoring or conventional US^7,11,20^. Semi-quantitatively calculated strain and elasticity ratios offer the potential benefit of more objective measurement methods, which correlate with the tissue’s elasticity characteristics. However, the reported strain or elasticity ratios varied with the study population and the specific elastography machine used. In our study, the mean strain ratio and elasticity ratios of benign and malignant lesions were 2.275 ± 0.998 and 3.936 ± 1.666, respectively, on strain elastography, and 2.576 ± 2.443 and 8 ± 8.55, respectively, on shear wave elastography (*P* < 0.001). In this study, the mean strain ratio of malignant masses was lower than those determined in previous studies by Mu *et al*., Zhi *et al*., and Cho *et al*. with the EUB-8500, 900, and HV-900 machines (Hitachi Medical, Tokyo, Japan) respectively; Alhabshi *et al*. with the Philips iU22 (Philips Healthcare, Bothell, WA, USA); and Fausto *et al*. with the Logic EQ (GE Healthcare, Milwaukee, USA)^9,13,20–22^. Our study population excluded patients with palpable lesions, the mass’ mean size was < 2.5 cm, and 13% of the lesions were of the DCIS type, potentially explaining the disparity between the results of our study and those aforementioned.

Although strain and elasticity ratios are objective semi-quantitative methods, the reported cut-off values also vary according to the study population and the machine used. Mu *et al*., Zhi *et al*., and Cho *et al*. calculated fat strain ratio cut-off values of 3.01, 3.05, and 2.24, respectively, with the EUB-8500, 900, and HV-900 machines, respectively; Alhabshi *et al*. calculated a value of 5.6 with the Philips iU22; and Fausto *et al*. calculated a value of 3.3 with the Logic EQ (GE Healthcare, Milwaukee, USA)^9,13,20–22^. Ng *et al*., Olgun *et al*., and Au *et al*. calculated elasticity ratio cut-off values of 2.2, 4.70, and 3.56, respectively, with the Aixplorer machine (Supersonic Imagine, Aix en Provence, France)^23–25^. In our study, we determined cut-off values of 2.93 for strain ratio, and 4 for elasticity ratio. Further technological development is needed to achieve standardization of elastography and provide semi-quantitative and quantitative methods.

When lesions were downgraded from BI-RADS category 4a to 3 by the cut-off value of max. kPa = 36.1, specificity increased significantly without significant loss of sensitivity in all four readers. Considering previously published studies of shear wave elastography, this cut-off value falls between previously reported values. In a study by Kim *et al*., the maximum elasticity (Emax) value, with a cut-off of 87.5 kPa, had the highest AUC value^26^. By using the optimal cut-off value (87.5 kPa), they were able to reduce 75.5% of unnecessary biopsies in BI-RADS category 4a lesions, but missed five malignancies, so they favoured the conservative strategy (Emax <20 kPa) so that no malignancies would be downgraded to category 3^25^. In a study by Lee *et al*., the conservative strategy of downgrading BI-RADS category 4a masses with a maximum elasticity <30 kPa was deployed^5^. Ng *et al*. determined the cut-off value of Emax to be 56 kPa^24^. “Blue” or “soft” cancers are occasionally found on shear wave elastography^10^. In this study, two cases of DCIS showed as blue on shear wave elastography.

Our study had several limitations. First, the study population consisted of patients undergoing diagnostic examination of a known mass and were scheduled for biopsy, and who, therefore, did not represent the screening population. Second, this was a single-site prospective study, but radiologists reviewed acquired static conventional US and shear wave elastography images in an independent manner. Although they reviewed video of conventional US, this does not emulate with absolute accuracy real-time US. Third, measurement of quantitative values was performed by one radiologist, and the interobserver variability of acquiring quantitative values was not evaluated. Further, the cut-off points of strain and elasticity ratios might change with the size and location of the reference fat region, which would require additional investigation. Fourth, direct comparison of performance of the visual score and strain or elasticity ratios was not possible, as the visual scores were four different values obtained by four different readers; conversely, strain and elasticity ratio were derived as a single-measured value by the elastography equipment. Therefore, we could only compare 95% CIs. Fifth, as this study only included patients who agreed to participate and those with non-palpable lesions, the study population was relatively small; thus, our results are not representative of all histologic types of breast lesions. Sixth, the ultrasonography follow-up period of the benign lesions was shorter than the conventional two-year follow-up period due to the short study duration. However, all patients were followed up for >12 months. In addition, multivariate analysis for evaluating various factors was not performed. Therefore, for our results to be clinically useful, a large prospective study should be performed for confirmation.

In conclusion, adding either strain or shear wave elastography improved the diagnostic performance of conventional ultrasonography. The cut-off values showing the best diagnostic performance for strain ratio, elasticity ratio, and the colour scores for strain and shear wave elastography were 2.93, 4, 3, and 2, respectively.

## Methods

### Patients

This prospective study was approved by the institutional review of Seoul National University Bundang Hospital of Seoul National University (IRB No. 1412/278-001), and all participants signed informed consent forms. All experiments were performed in accordance with relevant guidelines and regulations. From March 2015 to May 2015, percutaneous breast biopsies were performed in patients with lesions of Breast Imaging Reporting and Data System (BI-RADS) category 4 or 5 and in patients with masses of BI-RADS category 3 who wanted biopsy confirmation. Breast biopsies were conducted for 344 masses in 312 patients at our institution. Among these, 163 masses were excluded because the patients refused to participate in the study, 61 were excluded because they were palpable, and 12 were excluded because one-year follow-up data following a benign biopsy result were not available. Finally, 108 non-palpable lesions, from 94 patients (mean age, 48.7 years; age range, 23–77 years), were evaluated with ultrasound elastography before percutaneous biopsy. Subsequently, a dedicated breast radiologist performed the biopsies via a 14-G semi-automated core biopsy needle with a STERICUT^®^ device (TSK Laboratory, Tochigi, Japan) under US guidance.

We excluded patients <18 years of age, who had bleeding disorders, or lesions not visible on US.

### Image acquisition

One radiologist (13 years’ experience in breast imaging) performed conventional B-mode sonography with a 3–12-MHz linear transducer (RS80A with Prestige, Samsung Medison, Co. Ltd., Seoul, Korea) for 108 lesions. For each, transverse and longitudinal static images and video clips were obtained. The device settings for grayscale imaging using the breast setting were as follows: 25–50% gain, with compound imaging, 90–100% power, and an appropriated adjusted focal zone set according to lesion depth. A video clip was obtained. The maximum diameter of each lesion was measured along the longest axis of the mass on the US images.

The same radiologist also performed US elastography of the 108 lesions, using a 3–12-MHz linear transducer (RS80A, Samsung Medison Co., Ltd, Seoul, Korea) to obtain strain images, and a 6–13-MHz linear-array transducer from the Aixplorer US system (Supersonic Imagine, Aix en Provence, France) to obtain shear wave images, with both transverse and longitudinal scans. Elastographic images were generated using freehand manual compression by applying the transducer very lightly, as described by Itoh *et al*.^1^, perpendicular to the chest wall, with the position of the pectoralis muscle parallel to the probe. The dedicated software (ElastoScan TM, Samsung Medison) provided feedback regarding use of adequate compression and elastographic quality. Since tissue becomes stiffer when compressed^27^, we avoided compression during shear wave elastography (ShearWave TM, Supersonic Imagine) to prevent artefact generation. For elastography, the same depth, focus position, and gain settings were used as during conventional imaging. For each lesion, colour-scale strain images, colour-scale shear wave images with kPa, the lesion-to-fat strain ratio (i.e., strain ratio), and lesion-to-fat elasticity ratio (i.e., elasticity ratio) were obtained.

**Figure 4:**
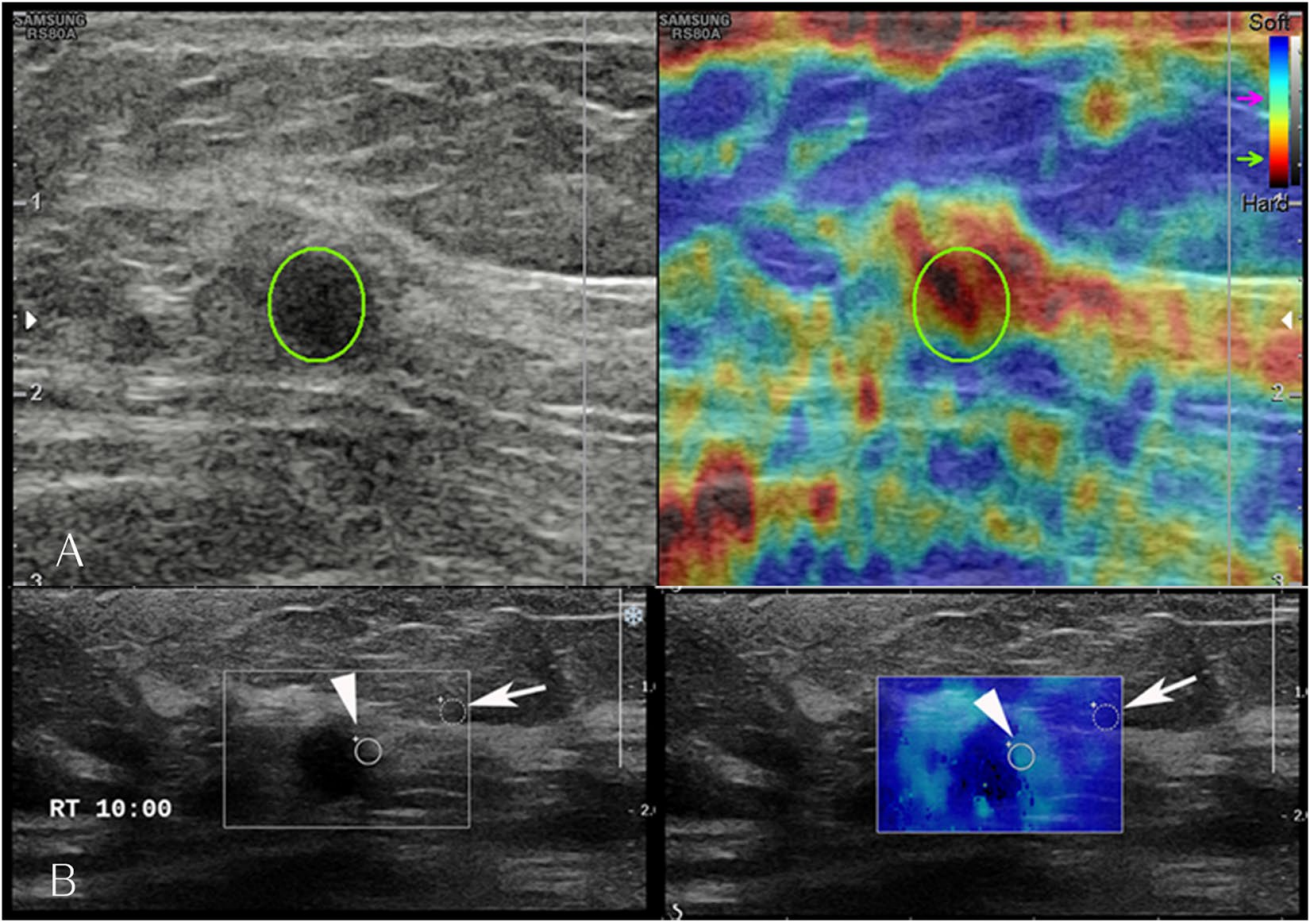
Representative images showing the region of interest used to obtain the strain ratio and elasticity ratio. (**A**) An oval region of interest (ROI) was set to include the mass (circle), in which the ultrasound machine automatically calculated and visualized the strain ratio as the mean strain within the ROI drawn along the border of the mass divided by the mean strain of the fat located at and above the level of the ROI set for mass strain measurement; the strain measured within the ROI set for mass measurement was excluded. (**B**) The elasticity ratio was calculated as the target round, solid ROI, located within the stiffest part of the lesion (arrow head) divided by another reference ROI in the adjacent subcutaneous fat (arrow).

To calculate the strain and elasticity ratios, the regions of interest (ROI) were placed in the hardest portion of lesions on the colour-scale images. Colour images from both elastography systems were presented in split-screen mode with grayscale images. Real-time elastography was visualized using a 256-colour mapping scale corresponding to the degree of strain; blue represented stiff tissue (i.e., least strain) and red represented soft (i.e., greatest strain). In strain elastography the reference fat region around a given lesion ROI was automatically identified at the level of or above the lesion ROI while the mean strain of the reference was calculated as the mean strain of the identified fat region^28^ (Fig. 4). In contrast, in shear wave elastography, the reference fat region around a given lesion ROI was manually drawn by the examiner who placing a 2-mm circle at the fat layer closest to the lesion ROI^8^ (Fig. 4).

The strain and elasticity ratios were obtained both in transverse and longitudinal scans. When the two scans showed similar image quality, the higher ratio value were selected; when the two scans showed different image quality, the ratio value of the higher quality image was selected.

### Image analysis

Four radiologists, each with >3 years’ experience in elastography, independently reviewed the images. The transverse and longitudinal grayscale and elastographic images were converted into sonographic images, then stored in a medical image storage and communications system (INFINITT PACS, INFINITT Healthcare, Seoul, Korea). A video clip of each mass’ grayscale image was stored in PACS. Each set of review images was arranged randomly in Microsoft Power Point. The reviewers were blinded to the final pathologic outcomes. At first, each lesion captured by conventional sonography with video was scored on a cancer probability scale of 0–100% and evaluated with BI-RADS (2–5) simultaneously. For receiver operating characteristic (ROC) curve analysis, we used the continuous cancer probability scale instead of BI-RADS, as suggested by Jian and Merz^29^. A score of 0 indicated a benign finding, similar to BI-RADS final assessment category 1–2^30^. A score of 1–2% indicated a “probably benign” finding, similar to BI-RADS final assessment category 3^30^. A score from 3–10% indicated “low suspicion of malignancy,” similar to BI-RADS final assessment category 4a^30^. A score from 11–50% indicated “moderate suspicion of malignancy,” similar to BI-RADS final assessment category 4b^30^. A score of 51–94% indicated “high suspicion of malignancy”, similar to BI-RADS final assessment category 4c^30^. Finally, a score from 95–100% was “highly suggestive of malignancy,” similar to BI-RADS final assessment category 5^30^.

After 2 and 4 weeks, each lesion was re-scored on the same cancer probability scale and re-evaluated by BI-RADS (2–5), while colour-scale strain images were additionally reviewed with conventional ultrasonography images. To evaluate the colour-scale images, the five-point Tsukuba score and elasticity score were recorded. The patients’ images were arranged randomly each time.

### Treatment and follow-up

In cases with discordant pathologic and radiologic findings, with a malignant or borderline (i.e., atypical ductal hyperplasia [ADH]) pathological diagnosis, and with benign lesions necessitating surgical resection (i.e., phyllodes tumours), further surgical biopsy was recommended. For cases in which the imaging and histologic diagnoses were benign, follow-up US was recommended at a 6-month interval.

### Statistical analysis

The clinical, radiological, and pathological data of the 108 patients were collected for statistical analysis. The diagnostic performance of colour-scale strain or shear wave images for multi-readers, based on cancer probability scale, was analysed by comparing the areas under the ROC curves using OR-DBM MRMC 2.4 (available from http://perception.radiology.uiowa.edu). Optimal cut-off values for strain ratio, elasticity ratio, and kPa in differentiating between benign and malignant breast lesions were determined using MedCalc (MedCalc Software, Mariakerke, Belgium). The 95% confidence intervals (CI) of the obtained cut-off values were compared to those of conventional images with or without additional review of colour-scale strain or shear wave images.

Interobserver agreement in colour score and BI-RADs was evaluated by the kappa coefficient using Stata (Version 13 IC; Stata-Corp, USA). Interobserver agreement in grading cancer probability was evaluated using the intraclass correlation coefficient (ICC) calculated with SPSS Statistics (version 19; IBM Corporation, Armonk, NY) software.

The diagnostic performance of each reader, based on hypothetically adjusted BI-RADS categories, was analysed by the McNemar test, using SPSS Statistics.

### Hypothetical adjustment of BI-RADS categories

Lesions initially graded as BI-RADS category 4a were hypothetically downgraded to BI-RADS category 3 if the reader gave a colour score of 1, 2, or 3 for strain or shear wave images, or the kPa, or strain or elasticity ratios were below the cut-off. After hypothetical adjustment, lesions with BI-RADS category ≥4a were coded as “malignant.” For colour score, the cut-off score with best performance was also determined.

### Data availability

The data are not available for public access because of patient privacy concerns, but are available from the corresponding author on reasonable request.
